# P-981. Bridging language divides and improving global health education for fellows: Implementation of a real-time closed captioned translation tool for an international case conference

**DOI:** 10.1093/ofid/ofae631.1171

**Published:** 2025-01-29

**Authors:** Sam Peterson, Scott Borgetti, Ryan D Knodle, Yori A Roque, David de luna, Rita A Rojas-Fermin, Alfredo J Mena Lora

**Affiliations:** University of Illinois at Chicago, Chicago, Illinois; University of Illinois at Chicago, Chicago, Illinois; University of Illinois at Chicago, Chicago, Illinois; Hospital Metropolitano de Santiago (HOMS), Santiago, Santiago, Dominican Republic; Internist-infectologist, Santiago de los cabelleros, Santiago, Dominican Republic; Hospital General de la Plaza de la Salud, Santo Domingo, Distrito Nacional, Dominican Republic; University of Illinois Chicago, Chicago, Illinois

## Abstract

**Background:**

Delivering a broad curriculum that includes diseases uncommon in the United States (US) such as tropical infections is of interest to program directors. Exposure to global health and tropical medicine is often limited by geographical and language barriers. To address this, our institution established an international case conference within the ID curriculum using Zoom's close-captioned translation technology.

**Figure:**
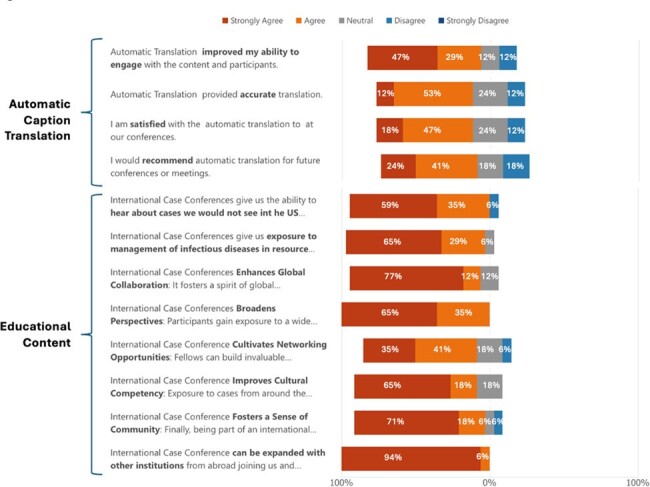

Attitudes and perceptions on automatic caption translation and the educational content of our international case conference

**Methods:**

A monthly conference was established in August 2021 with two US-based academic programs and 4 non-US based academic programs in the Dominican Republic (DR). This conference delivers 30-minute case presentations, with one case from the US and another from a DR site. Cases are chosen to reflect the unique pathologies encountered at respective sites. Conferences are via Zoom with an added automatic close-captioned translation feature. In March, an anonymous online survey was developed and sent to all participants to assess attitudes and perceptions on the educational content of the conference and effectiveness of the translation technology.

**Results:**

A total of 17 individuals participated in the survey, including 11 US faculty, 5 US fellows, and 1 physician assistant. All reported English as their primary language. The translation function showed 65% satisfaction and 77% of participants agreed it improved engagement (Figure 1). Most respondents were very satisfied (53%) or satisfied (41%) with the conference (Figure 2). The diversity of cases was satisfactory (47%) or very satisfactory (29%) by most respondents. Additionally, 94% agreed the conference broadened exposure to cases and management uncommon in the US, 88% agreed it fosters global collaboration, and 82% agreed it improved cultural competency (Figure 1). All participants agreed that the conferences broaden global perspectives.

**Figure:**
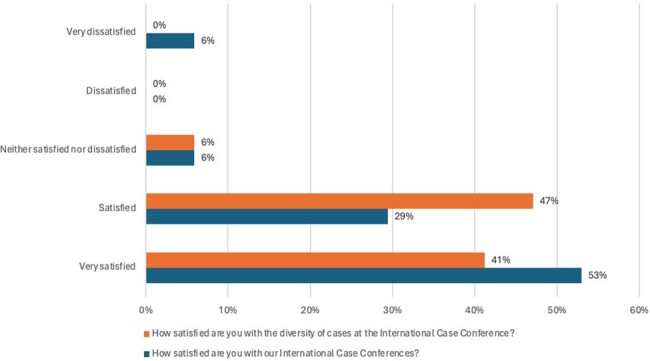

**Conclusion:**

We report a novel approach to integrating technology into medical education, aiming to enhance the learning by facilitating global collaboration and diversity of case discussions. The conference enables real-time multilingual discussions, enriching the learning experience with a diversity of cases and perspectives. This approach broadens the fellows' exposure and fosters international collaboration.

**Disclosures:**

**Scott Borgetti, MD**, GSK: Grant/Research Support **Rita A. Rojas-Fermin, MD,FIDSA**, Gilead: Advisor/Consultant|Pfizer: Advisor/Consultant|Pfizer: Honoraria

